# Supplementary material for: Detection of bisphenol A in thermal paper receipts and assessment of human exposure: A case study from Sharjah, United Arab Emirates
Source: PLoS One. 2023 Mar 28;18(3):e0283675. doi: 10.1371/journal.pone.0283675 (PMC10047534; doi:10.1371/journal.pone.0283675)
Supplement: Attachment — Submitted filename: Response to Reviewers.docx [file pone.0283675.s001.docx]

Detection of bisphenol A in thermal paper receipts and assessment of human exposure: A case study from Sharjah, United Arab Emirates

Lucy Semerjian, Najla Alawadhi, Khulud Nazer

**Response to Reviewers:**

The authors appreciate the Editor and reviewers for their time and efforts in reviewing the manuscript and providing valuable comments. The authors have carefully considered the comments and tried their best to address raised suggestions. We hope the manuscript after careful revisions meets your high standards. The authors welcome further constructive comments, if any. Below we provide the point-by-point responses to each reviewer. All modifications in the manuscript have been highlighted in Track Changes mode.

**REVIEWER 1:**

**Comment 1:** Abstract is vague, please rewrite to be clear for readers

**Response 1:** The abstract has been revised for further clarification and to incorporate also Comment 2 raised by Reviewer 3 below

**Comment 2:** Introduction is too long

**Response 2:** To find a balance between this comment and comment 1 raised by Reviewer 3 who expressed that Introduction is “too short”, we have revised the Introduction to read more scientifically and shortened few sentences as applicable. Thanks for your valuable comments.

**Comment 3**: Number of paper is insufficient

**Response 3:** The study area is Sharjah City (Population 1.275 million (2019)) and number of receipt samples was selected based on literature review and typical number of samples used in similar global studies. For example, 40 sampling location were selected by Khatun et al., 2022 in Dhaka, Bangladesh, 12 samples were collected in duplicates in New Delhi, India (ToxicsLink, 2012), 44 samples were collected from various regions in Belgium by Geens et al., 2012. All such locations have population counts much higher than Sharjah City thus authors adopted similar numbers of thermal paper samples in the current study.

**Comment 4:** Tables unclear, specially Table 3

**Response 4:** All Tables have been revised to comply by journal’s format and read clearer

**Comment 5:** Discussion must be supported by extra references

**Response 5:** Initially, discussion of results were done in comparison to findings from similar research conducted worldwide where the receipts were specifically collected from supermarkets, restaurants, ATMs and others to be comparable to the sources of thermal receipts in the current study. Additional discussion and references are now included and tabulated (Table 1 and 2) to expand and address the reviewer’s comment.

**REVIEWER 2:**

**Comment 1:** The conclusion should be more specific. It should only contain the important findings. The discussion section can include the comments made in the conclusion.

**Response 1:** The Conclusion section was revised to include specifically recorded findings. Additional comments and recommendations are moved to the Results and Discussion section as suggested by Reviewer. Thank you for your valuable comments.

**Comment 2:** It would enrich the study if the study participants' health problems attributable to BPA toxicity were investigated

**Response 2:** The authors agree that investigation of health impacts attributable to BPA are important yet the proposed aspect is out of scope of the current study as the study aims at investigating BPA levels in thermal receipts circulating in Sharjah City, UAE and conducting related risk assessments. Thus, human monitoring was not included in this study as it is subject to ethical approvals and securing relevant consents which are on the authors’ research agenda. We thank the reviewer on the valuable comment.

**Comment 3:** There are a few grammatical/typographical mistakes

**Response 3:** The manuscript was revised with best efforts to eliminate grammatical/typographical mistakes, where applicable.

**REVIEWER 3:**

The paper presents the results of studies Detection of Bisphenol A in Thermal Paper Receipts and Assessment of Human Exposure: A Case Study from Sharjah, United Arab Emirates. The issue raised in the paper is important and the manuscript falls within the scope of PLOS one. The paper would be of interest to the readers of the journal; however, I recommend this paper undergo major revisions. The results are quite interesting but still the flaws are needed to be addressed.

**Comment 1:** Introduction is too short and simple and written in very simple and unscientific way

**Response 1:** This comment, although in contradiction with Reviewer 1 Comment 2, was addressed by revising the Introduction to read more scientifically and shortened few sentences as applicable

**Comment 2:** I think that at the end of the abstract should be inserted a brief conclusion to help readers in getting a better understanding of what is the significance of this study. Quantitative information should be provided in the abstract.

**Response 2:** Quantitative information such as BPA levels, exceedances, and estimated daily intakes were made sure to be included in the abstract, and the “Abstract” section was concluded by a phrase highlighting the significance of the current study

**Comment 3**: According to many studies that have been done, it is better to compare the results of this study with other studies in the results section; this could be accompanied by adding a table at the end of the results to make the comparison easier and to determine the superiority of this study.

**Response 3**: Initially, discussion of results were done in comparison to findings from similar research conducted worldwide where the receipts were specifically collected from supermarkets, restaurants, ATMs and others to be comparable to the sources of thermal receipts in the current study. Additional discussion and references are now included and tabulated (Table 1 and 2) to expand and address the reviewer’s comment. Thank you for your valued comment.

**Comment 4:** Any final polishing of manuscript should be completed prior to submission and it is strongly recommended to check the text by professional language editing services to make respective grammar corrections, especially when English is not the Authors' native language.

**Response 4:** The manuscript was revised with best efforts to eliminate grammatical/typographical mistakes, where applicable.

**REVIEWER 4:**

**Comment 1:** I suggest to add "occupational exposure" as keyword; add information about number of respondent and human sampling method in abstract.

**Response 1:** “Occupational exposure” is added as a keyword. Human sampling was out of the scope of the current study which primarily aims at investigating BPA levels in thermal receipts circulating in Sharjah City, UAE and assessing related risks rather than investigating human subjects and conducting surveys. Thank you for your valuable comment

**Comment 2:** In line 45 - 46, PVC food packaging stretch films, and dental sealants, thermal paper is... change with "PVC food packaging stretch films, and dental sealants, thermal paper are..."

**Response 2:** Sentence (now line 54) corrected as suggested, thank you.

**Comment 3:** Add citation in statement in line 45 - 46. Why thermal paper is included in the major source of BPA.

**Response 3:** Citation included (now in line 54) as suggested

**Comment 4**: Add citation in statement in line 46 – 47. Explain about health effects from BPA exposure in general. Are there any epidemiological studies related BPA exposure from skin to human health problems?

**Response 4**: Additional citations are included as suggested. Health effects from BPA exposure are also discussed in now line 51. Thank you for your valuable comment

**Comment 5:** I suggest to display sampling area map in the manuscript. Why only 30 samples of thermal paper analyzed?

**Response 5:** The study area is Sharjah City (Population 1.275 million (2019)) and number of receipt samples was selected randomly based on literature review and typical number of samples used in similar global studies. For example, 40 sampling location were selected by Khatun et al., 2022 in Dhaka, Bengladesh, 12 samples were collected in duplicates in New Delhi, India (ToxicsLink, 2012), 44 samples were collected from various regions in Belgium by Geens et al., 2012. All such locations have population counts much higher than Sharjah City so authors adopted similar numbers of thermal paper samples in the current study. It is noteworthy to mention that higher number of samples would have been desired yet incur higher laboratory efforts and costs. Thank you for the valuable comment

**Comment 6:** There are 3 constant value for k and AF. can you describe what are for?

**Response 6:** Constant values for *k* and AF were selected from the extensive literature review conducted throughout the study. The authors made efforts to investigate all possible reported values for *k*, paper-to-skin transfer coefficients of BPA and all reported absorption fractions of BPA by skin (AF) as reported by Bernier and Vanderberg (2017) for a comprehensive risk assessment covering various scenarios.

**Comment 7:** Please change table's format according to PLOS one format

**Response 7:** Noted with thanks, all Tables have been revised to comply by journal’s format.

**Comment 8:** I think your suggestion of sanitizers and lotion application should be reduced is not accepted. Because it will contradict with other regulation related COVID 19 transmission prevention.

**Response 8:** Noted with thanks, the revised recommendation calls occupationally exposed persons to wear gloves, especially with sanitizers and lotion application during COVID-19 pandemic, to reduce exposure to BPA from thermal papers.
