# Supplementary material for: Detection of bisphenol A in thermal paper receipts and assessment of human exposure: A case study from Sharjah, United Arab Emirates
Source: PLoS One. 2023 Mar 28;18(3):e0283675. doi: 10.1371/journal.pone.0283675 (PMC10047534; doi:10.1371/journal.pone.0283675)
Supplement: Attachment — Submitted filename: Response to Reviewers.docx [file pone.0283675.s002.docx]

Detection of bisphenol A in thermal paper receipts and assessment of human exposure: A case study from Sharjah, United Arab Emirates

Lucy Semerjian, Najla Alawadhi, Khulud Nazer

**Response to Reviewers:**

The authors appreciate the Editor and reviewers for their time and efforts in reviewing again the manuscript and providing valuable comments. The authors have carefully considered the comments and hope the manuscript now meets your high standards.

Below we provide the point-by-point responses to each reviewer. All modifications in the manuscript have been highlighted in Track Changes mode.

**Reviewers' comments:**

**Reviewer #1:**

*No response/comments received*

**Reviewer #2:**

*Comment:* The reviewers’ comments are well addressed. However, it is preferable to keep the conclusion and recommendation separate.

*Response:* Thank you, as suggested the “Conclusions” have been separated from the “Recommendations” in the revised version

**Reviewer #3:**

*Comment:* Thanks for your correct revision, and the quality of the paper has been improved greatly so that it is adequate to publish in journal.

*Response:* Thank you for your positive response

**Reviewer #4:**

*Comment:* please to explain about 3 numbers of k (paper-to-skin transfer coefficient) and AF (absorption factor) that you display on table 3 - 7 ? why there are 3 numbers? what are they for? Is the number differentiated based on the age of the respondent or otherwise? you can add the information on the text or in table.

*Response:* Thank you. Further explanations for both *k* and AF, namely as below, were added in-text to the “Materials and Methods: BPA exposure assessment” section (p. 8, Lines 167-186, and 172-175 in Revised Manuscript with Track Changes).

For *k* values: Reported *k* values varied as conducted extractability experiments exhibited varying coefficients based on skin conditions being dry, wet, or very greasy.

For AF values: A wide range of absorption factors have been reported in previous studies as they are calculated from different experimental systems: 2.3–8.6% from skin explants, and 27% from live hands after application of BPA

*Comment:* TDI is Tolerable Daily Intake not Tolerable day intake (please review the text).

*Response:* The text has been reviewed, the phrase now reading Tolerable Daily Intake (p. 14, Line 253 in Revised Manuscript with Track Changes)
